# Prevalence of and risk for gastrointestinal bleeding and peptic ulcerative disorders in a cohort of HIV patients from a U.S. healthcare claims database

**DOI:** 10.1371/journal.pone.0180612

**Published:** 2017-06-30

**Authors:** Emily Bratton, Vani Vannappagari, Monica G. Kobayashi

**Affiliations:** 1 Real World Evidence Solutions, QuintilesIMS, Durham, North Carolina, United States of America; 2 Epidemiology and Real World Evidence, ViiV Healthcare Ltd, Research Triangle Park, North Carolina, United States of America; 3 Department of Epidemiology, University of North Carolina-Chapel Hill, Chapel Hill, North Carolina, United States of America; 4 PAREXEL Access Consulting, PAREXEL International, Durham, North Carolina, United States of America; University Hospital Llandough, UNITED KINGDOM

## Abstract

The primary study objectives were to estimate the frequencies and rates of gastrointestinal bleeding and peptic ulcerative disorder in HIV-positive patients compared with age- and sex-matched HIV-negative subjects. Data from a US insurance claims database was used for this analysis. Among 89,207 patients with HIV, 9.0% had a GI bleed, 1.0% had an upper gastrointestinal bleed, 5.6% had a lower gastrointestinal bleed, 1.9% had a peptic ulcerative disorder diagnosis, and 0.6% had both gastrointestinal/peptic ulcerative disorder. Among 267,615 HIV-negative subjects, the respective frequencies were 6.9%, 0.6%, 4.3%, 1.4%, and 0.4% (*p*<0.0001 for each diagnosis subcategory). After combining effect measure modifiers into comedication and comorbidity strata, gastrointestinal bleeding hazard ratios (HRs) were higher for HIV-positive patients without comedication/comorbidity, and those with comedication alone (HR, 2.73; 95% confidence interval [CI], 2.62–2.84; HR, 1.59; 95% CI, 1.47–1.71). The rate of peptic ulcerative disorder among those without a history of ulcers and no comorbidity/comedication was also elevated (HR, 2.72; 95% CI, 2.48–2.99). Hazard ratios of gastrointestinal bleeding, and peptic ulcerative disorder without a history of ulcers were lower among patients infected with HIV with comedication/comorbidity (HR, 0.64; 95% CI, 0.56–0.73; HR, 0.46; 95% CI, 0.33–0.65). Rates of gastrointestinal bleeding plus peptic ulcerative disorder followed a similar pattern. In summary, the rates of gastrointestinal/peptic ulcerative disorder events comparing HIV-infected subjects to non–HIV-infected subjects were differential based on comorbidity and comedication status.

## Introduction

Gastrointestinal (GI) symptoms including diarrhea, nausea, vomiting, difficulty swallowing, weight loss, and abdominal pain are among the most frequently encountered symptoms in HIV/AIDS[1]. Before the introduction of combination antiretroviral therapy (cART), GI conditions were common (50%-90%) among patients with AIDS, and were mainly a result of opportunistic infection[2]. Despite the increasingly widespread use of highly active antiretroviral therapy in HIV infection, the GI tract is still frequently affected by HIV-associated disease processes[3]. Esophageal and gastric lesions are primarily caused by candidiasis, cytomegalovirus (CMV) infection, herpes simplex virus infection, and idiopathic ulceration[3]. Cytomegalovirus remains the most common opportunistic gastric infection (25% of lower GI bleeding events, 14% of endoscopic diagnoses) in HIV-positive patients in the cART era[4,5]; it is the most frequently identified cause of ulcer disease in symptomatic patients[3,6]. With widespread use of combination antiretroviral therapy, the incidence of opportunistic disorders among patients with HIV have decreased dramatically. One study noted a significant declining trend in opportunistic infections of the GI tract (69%-13%), with an associated rise in the prevalence of non-opportunistic GI problems (31%-87%)[5].

Gastrointestinal bleeding is a rarer GI symptom compared with nausea, vomiting, and diarrhea (roughly half or more of all patients with HIV experience the latter symptoms)[7,8]. Post–highly active antiretroviral therapy incidence data for GI bleeding are limited. Data generally show rates of GI bleeding ranging from 1% to 14%[9–11]. Two studies reported rates for upper GI bleeding around 1%[9–11]. Upper GI bleeding occurs about 3 times as often as lower GI bleeding (10% vs 3% in 1 study)[12]. Among patients with AIDS, GI bleeding can be the result of HIV-related causes such as CMV, Kaposi sarcoma, non-Hodgkin’s lymphoma, and herpes simplex virus, among others. Upper GI bleeding can also be the result of non–HIV-related causes such as a peptic ulcer, portal hypertension, or a Mallory-Weiss tear[12,13].

Very few studies have examined peptic ulcerative disorder (PUD) among HIV-infected populations in the cART era. Peptic ulceration is less common in HIV-infected adults than in uninfected adults, despite the high prevalence of *Helicobacter pylori* in low- and middle-income countries, thought to be attributable to a lack of/reduced stomach acid[14]. Peptic ulcerative disorder tends to have a chronic and remitting course with imperfect correlation between symptoms and the presentation of ulcers, which makes estimating incidence and prevalence difficult. In the general population, most ulcers (20%-40%) can be attributed to infection with *H*. *pylori* and non-steroidal anti-inflammatory drug (NSAID) use[15]; other causes include the use of antiplatelet agents, stress, cigarette smoking, diet, genetics, infection with *Helicobacter heilmanii*, CMV infection, Behcet disease, Zollinger-Ellison syndrome, Crohn’s disease, and cirrhosis with portal hypertension[16].

The primary goals for this analysis were to estimate the frequencies and rates of GI bleeding and PUD in HIV-positive patients relative to age- and sex-matched HIV-negative patients. This study updates and builds upon a previous analysis[11] that examined GI bleeding among patients with HIV in the same US healthcare claims database (1997–2008), where only univariate statistics were presented. Here, we expand our comparison to include the HIV-negative comparator population and estimate-adjusted hazard rate ratios (HR) for the recent cART era (2005–2013).

## Methods

### Data source

This was a matched, retrospective cohort study comparing HIV-positive and HIV-negative patients with GI bleeding and/or PUD. Patient demographic information (including age and sex) and outcomes data from the Clinformatics^™^ DataMart Multiplan (IMPACT) database, a product of OptumInsight Life Sciences, Inc (Eden Prairie, MN) and a comprehensive, de-identified US healthcare claims database consisting of over 118 million unique patient records, were used for analysis. Cases of any GI bleeding and PUD among HIV-positive patients from January 1, 2005 through March 31, 2013 were identified using the International Classification of Diseases, Ninth Revision (ICD-9 Codes).

### Study population

HIV-positive patients were compared with age- and sex-matched HIV-negative patients (matching ratio, 1:3). All HIV-positive patients at least 18 years of age in the IMPACT database who were continuously enrolled with full pharmacy benefits for at least 6 months during the study period were included in the analysis. Those with a gap of more than 1 month in their enrolment were excluded from the analysis. The comparator group consisted of age- and sex-matched patients at least 18 years of age without a diagnosis code for HIV. HIV-positive patients were defined as those having a code for HIV (ICD9 042and/or V08). All patients infected with HIV must have been diagnosed with HIV prior to the diagnosis of the GI disorder under consideration.

### Data analysis

Baseline demographic and clinical characteristics were tabulated using distribution frequencies and Chi-square significance tests to compare HIV-positive patients and HIV-negative patients. Frequencies of relevant disease conditions, comedications, and ART medications were examined. Drug exposure was tabulated at 1 month and 3 months prior to having a GI/PUD diagnosis.

Hazard ratios were estimated to determine the rates of primary episodes of the following outcomes among HIV-positive patients relative to the matched HIV-negative group: (a) any GI bleeding, (b) PUD, and (c) PUD plus any GI bleeding. Potential risk factors for GI bleeding and PUD evaluated for inclusion in the final, adjusted HR models predominantly fell into the categories of demographics (age, sex), comorbidities (*H*. *pylori*, hepatitis C virus, hepatitis B virus, alcoholism with or without Mallory Weiss Tears, cirrhosis, other bleeding disorders), comedications (see next paragraph, below), and history of GI bleeding and/or PUD.

Comedications evaluated for effect measure modifiers (EMM) and confounding factors for HIV and GI bleeding included NSAIDs (aspirin and non-aspirin [NA]-NSAIDs separately), NSAIDs in high doses, oral corticosteroids (namely prednisone), selective serotonin reuptake inhibitors (SSRIs), opioids, and anticoagulants; for HIV and any PUD: NSAIDs, corticosteroids, cyclooxygenase (COX) inhibitors, prostaglandin analog (misoprostol), opioids and meclofenamic acid, oxaprozin, and tolmetin. Dosing was categorized as a “high” (>) or “low” (≤) dose for aspirin and NA-NSAIDs (Table 1). Comedication use was categorized as follows:

1. Exposed, had GI/PUD event: ≥10 days prescription coverage for comedication ±30 days of the event.
2. Exposed, did not have GI/PUD event: ≥10 days prescription coverage for comedication ±30 days of first comedication use.
3. Unexposed, had GI/PUD event: <10 days exposure ±30 days of the event; or had event and ≥10 days of exposure, but >30 days before the event.
4. Unexposed, did not have GI/PUD event: <10 days exposure ±30 days of first comedication use; or did not have ≥10 days exposure to comedication at all.

**Table 1:** Cutoff doses (mg/day) for NA-NSAIDs and aspirin (≤ low-dose or >high-dose).

NSAID	Daily dose, mg/day
**Aspirin**	300
**Aceclofenac**	100
**Acemetacin**	120
**Azapropazone**	600
**Diclofenac**	100
**Diflunisal**	1500
**Etodolac**	400
**Fenbufen**	900
**Fenoprofen**	1200
**Fenoprofen**	1200
**Flurbiprofen**	150
**Ibuprofen**	1200
**Indomethacin**	75
**Ketoprofen**	150
**Ketorolac**	90
**Mefenamic acid**	1000
**Meclofenamic acid**	300
**Meloxican**	7.5
**Nabumetone**	1000
**Naproxen**	750
**Oxaprozin**	1200
**Piroxicam**	20
**Sulindac**	200
**Tenoxicam**	10
**Tiaprofenic**	450
**Tolumetin**	1820
**Celecoxib**	200
**Etoricoxib**	90
**Rofecoxib**	25
**Valdecoxib**	40

NA-NSAID, non-aspirin non-steroidal anti-inflammatory drug.

All analyses were performed using SAS^®^ (v 9.3; SAS Institute, Inc, Cary, NC). Conditional Cox proportional-hazard models were used to estimate HRs; EMMs were checked for significance using *p*<0.05 from the log-rank statistic for tests of equality over strata; then assessed using *p*<0.05 for the interaction terms in the adjusted model. A backwards elimination strategy for assessing confounding variables was used with the log-likelihood ratio test (*p* <0.05) and ln-ln survival plots were generated to check that the proportional hazards assumptions were upheld.

### Ethics statement

The Clinformatics^™^ DataMart Multiplan (IMPACT) database is anonymized and contains no personal information.

## Results

There were 89,207 HIV-positive patients eligible for analysis, matched to 267,261 HIV-negative patients (Table 2). Most patients in both groups were male (73%) and about one-third were aged 40 to 49 years. Presence of comorbidity/coinfection and use of comedications were significantly more common among patients infected with HIV compared with patients uninfected with HIV. Prevalence of any GI bleeding among HIV-positive patients (9.0%) was significantly higher compared with HIV-negative patients (6.9%). Peptic ulcerative disorder was less common in both groups, but still significantly different (HIV-positive, 1.9%; HIV-negative, 1.4%; *p*<0.0001), and less than 1% had both GI bleeding and PUD in both groups. The majority of patients infected with HIV with lab values available (*n* = 1036) were virally suppressed; 43% had a CD4 cell count >500 cells/mm^3^ and 81% had a viral load ≤500 copies/mL.

**Table 2:** Demographic and baseline characteristics of age- and sex-matched HIV-positive and HIV-negative patients.

Characteristic		Patient group				
		HIV-positive		HIV-negative		
		n	%	n	%	*p*-value
**N**		89,207	(100)	267,621	(100)	
**Sex**						
	Male	65,393	(73.3)	196,179	(73.3)	
	Female	23,814	(26.7)	71,442	(26.7)	
**Age group, y**						
	18–29	18,075	(20.3)	54,225	(20.3)	
	30–39	26,026	(29.2)	78,078	(29.2)	
	40–49	29,478	(33.0)	88,434	(33.0)	
	50–59	12,635	(14.2)	37,905	(14.2)	
	60–69	2460	(2.8)	7380	(2.8)	
	70–79	533	(0.6)	1599	(0.6)	
**Comorbidity/Coinfection**^a^						<0.0001
	0 conditions	76,426	(85.7)	258,166	(96.5)	
	1 condition	9838	(11.0)	8489	(3.2)	
	2–3 conditions	2810	(3.2)	931	(0.4)	
	4–5 conditions	133	(0.2)	35	(0.0)	
**Comedications**						<0.0001
	0 medications	9164	(10.3)	53,643	(20.0)	
	1 medication	4186	(4.7)	22,335	(8.4)	
	2–3 medications	10,078	(11.3)	43,246	(16.2)	
	4–5 medications	10,547	(11.8)	34,050	(12.7)	
	>5 medications	55,232	(61.9)	114,347	(42.7)	
**Primary outcomes**						
**Any GI bleeding**						<0.0001
	Yes	7990	(9.0)	18,460	(6.9)	
	No	81,217	(91.0)	249,161	(93.1)	
**Upper GI bleeding**						<0.0001
	Yes	847	(1.0)	1692	(0.6)	
	No	88,360	(99.1)	265,929	(99.4)	
**Lower GI bleeding**						<0.0001
	Yes	5014	(5.6)	11,399	(4.3)	
	No	84,193	(94.4)	256,222	(95.7)	
**PUD**						<0.0001
	Yes	1664	(1.9)	3652	(1.4)	
	No	87,543	(98.1)	263,969	(98.6)	
**GI bleeding and PUD**						<0.0001
	Yes	505	(0.6)	1082	(0.4)	
	No	88,702	(99.4)	266,539	(99.6)	
**History of ulcer diagnosis**						<0.0001
	Yes	1049	(1.2)	2104	(0.8)	
	No	88,158	(98.8)	265,517	(99.2)	
**History of GI diagnosis**						<0.0001
	Yes	4520	(5.1)	9290	(3.5)	
	No	84,687	(94.9)	258,331	(96.5)	
**ART**^b^^,^^c^						
	Ever	50,411	(56.5)			
	Never	38,796	(43.5)			
**Lab values**^b^^,^^d^						
	Available	1036	(1.2)			
	Not available	88,171	(98.8)			
**CD4 count (cells/mm**^**3**^**)**^b^^,^^d^						
	<200	173	(20.4)			
	200-<350	148	(17.4)			
	350–500	166	(19.6)			
	>500	362	(42.6)			
**Viral load (copies/mL)**^b^^,^^d^						
	≤500	271	(81.4)			
	501–9000	30	(9.0)			
	10,000–29,999	10	(3.0)			
	≥30,000	22	(6.6)			

ART, antiretroviral; GI, gastrointestinal; PUD, peptic ulcerative disorder.

^a^*H*. *pylori*, other bleeding disorders, cirrhosis, alcoholism, hepatitis B, or hepatitis C.

^b^Among patients infected with HIV only.

^c^On or after HIV diagnosis; during study period January 1, 2005, to March 31, 2013.

^d^Within 1 month of GI or PUD diagnosis. Available labs are CD4 cell count or viral load.

Among patients with HIV with ART information and who had any GI/PUD event (*n* = 8945), 60.1% (*n* = 5378) had used ART. There were 4349 patients exposed to ART within 1 month of having an event, and 4790 patients within 3 months. Among patients with HIV with a GI/PUD event, any ART use according to outcome category ranged between 78% and 82% (88%-91% used ART within 3 months). Nucleoside reverse transcriptase inhibitors (NRTIs) were the most commonly prescribed ART class. The most frequent medications used were Truvada^®^ (emtricitabine/tenofovir; Gilead Sciences, Foster City, CA) and Atripla^®^ (efavirenz/emtricitabine/tenofovir disoproxil fumarate; Bristol-Myers Squibb, New York, NY and Gilead Sciences). The most common non-ART comedications taken within 1 month of a GI/PUD event included gastrointestinal/laxatives, anti-infective drugs, vascular agents, and psychotherapeutics. The drug classes used to report these comedications are the uniform system of classification (USC), developed by IMS Health (Danbury, CT) and the America Pharmaceutical Marketing Research Group (details available online at: Imshealth.com).

There were 2434 (27.2%) HIV-positive patients and 2288 (11.1%) HIV-negative patients who had at least 1 comorbidity and had a GI/PUD event (p<0.0001). Hepatitis C was the most prevalent across all outcome categories among patients infected with HIV, ranging between 7.4% and 18.5%. The most prevalent comorbidity among HIV-negative patients prior to a GI/PUD event varied; *H*. *pylori* was most common among patients with PUD (1.6%), and alcoholism and other bleeding disorders were most prevalent in the other GI/PUD outcome categories (0.9%-5.6%).

Crude HR model estimates had higher relative HRs for all GI/PUD events among patients infected with HIV relative to patients uninfected with HIV (HR_GI bleed_, 2.2; 95% confidence interval [CI], 2.2–2.3; HR_PUD_, 2.3; 95% CI, 2.1–2.4; HR_GI bleed and PUD_, 2.3; 95% CI, 2.1–2.6). Based on the *a priori* modeling strategy, there were numerous EMMs for each model. To provide meaningful estimates of the minimal adjustment set with fewer strata, composite variables of EMMs falling into either comorbidity or comedication categories were created; if a subject had any of the EMMs, then they were classified as “1” or “yes” for the corresponding composite variable. Univariate associations of these EMMs are shown in Table 3.

**Table 3:** Univariate associations of effect measure modifiers utilized in hazard rate models.

		HIV-positive		HIV-negative		Univariate *p*-value for interaction term	*p*-value for interaction term in full model
		n	%	n	%		
**GI bleeding**							
	H. pylori	889	1.00	966	0.36	<0.0001	<0.0001
	HCV	6142	6.89	1006	0.38	<0.0001	<0.0001
	Alcoholism	1757	1.97	1937	0.72	<0.0001	<0.0001
	Cirrhosis	1247	1.40	523	0.20	0.0003	0.0287
	HBV	4073	4.57	445	0.17	<0.0001	0.0013
	Low-dose NSAID	3476	3.90	6219	2.32	0.0006	0.0011
	High-dose NSAID	11,665	13.08	23,683	8.85	<0.0001	<0.0001
	Antiplatelet	1402	1.57	2314	0.86	<0.0001	<0.0001
	Anticoagulant	1877	2.10	2615	0.98	0.0297	0.0012
	Corticosteroid	4491	5.03	7264	2.71	0.0352	0.0030
	SSRI	12,396	13.90	16,186	6.05		<0.0001
	Opioids	11,882		16,815	6.28	<0.0001	<0.0001
	History of GI bleed	2756	3.09	8196	3.06	<0.0001	0.0004
**PUD**							
	*H*. *pylori*	889	1.00	966	0.36	0.0548	0.0231
	HCV	6142	6.89	1006	0.38	0.0004	0.0217
	Alcoholism	1757	1.97	1937	0.72	<0.0001	0.0003
	Cirrhosis	1247	1.40	523	0.20	0.0002	0.0039
	HBV	4073	4.57	445	0.17	0.0421	0.0161
	Low-dose NSAID	3878	4.35	6796	2.54	0.0275	0.0401
	Antiplatelet	1545	1.73	2497	0.93	0.0247	0.0052
	SSRI	13,370	14.99	17,198	6.43	<0.0001	<0.0001
	Anticoagulant	2077	2.33	2865	1.07	0.2929	0.0073
	Opioids	12,983	14.55	18,052	6.75	<0.0001	<0.0001
**GI bleeding + PUD**							
	*H*. *pylori*	889	1.00	966	0.36	0.0027	0.0018
	HBV	4073	4.57	445	0.17	0.4478	0.0212
	SSRI	13,552	15.19	17,415	6.51	0.0094	0.0001
	Antiplatelet	1577	1.77	2531	0.95	0.0164	0.0003
	Opioids	13,210	14.81	18,331	6.85	0.0012	0.0047

GI, gastrointestinal; HBV, hepatitis B virus; HCV, hepatitis C virus; NSAID, non-steroidal anti-inflammatory drug; PUD, peptic ulcerative disorder; SSRI, selective serotonin reuptake inhibitors.

All 4 adjusted HR models, stratified by EMMs (comorbidities, comedications), showed significantly increased rates of GI/PUD outcomes for patients infected with HIV relative to patients uninfected with HIV among those *without* comorbidities and comedications (Table 4). Three models showed a significantly decreased HR for patients infected with HIV relative to patients uninfected with HIV among patients infected with HIV with *both* comorbidities and comedications. Models stratified by either comorbidity or comedication only were mixed, but the majority lacked significance (Table 4). The GI bleeding model among subjects without a history of a GI bleed was significantly elevated among subjects in comedication and comorbidity strata (HR, 2.04; 95% CI, 1.86–2.24; and HR, 1.31; 95% CI, 1.14–1.52, respectively); PUD was significantly elevated among patients with comedication exposure (HR, 1.60; 95% CI, 1.29–1.97). In the GI bleeding with PUD model among subjects with comorbidities only, the HR was significantly protective (HR, 0.27; 95% CI, 0.11–0.67).

**Table 4:** Conditional cox proportional-hazard models of HIV infection status and GI bleeding, PUD, or GI bleeding and PUD Events; stratified by effect measure modification using composite comorbidities and comedications determined individually for each of the 3 outcomes.

	Neither comorbid nor comedication		Comedication only		Comorbid only		Comorbid AND Comedication	
	HR	(95% CI)	HR	(95% CI)	HR	(95% CI)	HR	(95% CI)
GI bleeding, without history of GI bleeding^a^	3.32	(3.17, 3.49)	2.04	(1.86, 2.24)	1.31	(1.14, 1.52)	0.81	(0.69, 0.94)
GI bleeding, with history of GI bleeding^a^	6.70	(3.11,14.44)	0.77	(0.26, 2.34)	3.18	(0.60, 16.89)	0.37	(0.06, 2.14)
PUD^b^	3.79	(3.40, 4.23)	1.60	(1.29, 1.97)	0.83	(0.62, 1.11)	0.35	(0.25, 0.49)
GIB and PUD^c^	3.85	(3.13, 4.74)	1.20	(0.82,1.76)	0.27	(0.11, 0.67)	0.08	(0.03, 0.22)

CI, confidence interval; GI, gastrointestinal; GIB, gastrointestinal bleeding; HR, hazard rate ratio; PUD, peptic ulcerative disorder.

^a^Comorbidity variable created from of *H*. *pylori*, HCV, alcoholism, cirrhosis, and HBV; comedication variable created from high- and low-dose NSAIDs, antiplatelet use, anticoagulant use, corticosteroid use, SSRIs, and opioid use. Model is adjusted for confounding variables by comedications, comorbidities, history of ulcers, high-dose aspirin, and prostaglandin use.

^b^Comorbidity variable created from of *H*. *pylori*, HCV, alcoholism, cirrhosis, and HBV; comedication variable created from low-dose NSAID use, SSRIs, antiplatelet use, and anticoagulant use. Model is adjusted for confounding variables by comorbidity, comedication, history of GI bleeding, history of ulcers, high-dose NSAID use, and corticosteroids use.

^c^Comorbidity variable created from *H*. *pylori* and HBV; Comedication variable created from SSRIs, antiplatelet, and opioid use. Model is adjusted for confounding variables by comorbidity, comedication, history of ulcers, history of GI bleeding, high-dose NSAID use, alcoholism, cirrhosis, and corticosteroid use.

## Discussion

The results of this study showed GI bleeding and PUD frequencies were significantly higher among HIV-positive patients. There was also an increased underlying prevalence of risk factors for GI bleeding and PUD among patients infected with HIV compared with subjects uninfected with HIV. Previous study results are highly variable based on the underlying study population, study size, and data sources and span different HIV treatment time periods (pre-cART, early and recent cART eras). Levels of diagnostic criteria and depth of risk factor information are also contributors to study differences. This is the first large, US claims database cohort study to estimate rates of GI bleeding and PUD among patients with HIV in the recent cART era.

In this study, the prevalence of any GI bleeding, upper GI bleeding, and lower GI bleeding among HIV-positive patients were 9.0%, 1%, and 5.6%, respectively. In a prior analysis using the same database (1997–2008), the frequency of GI bleeding among patients infected with HIV was 7.8%, and the frequencies of upper and lower GI bleeding, specifically, were 0.8% and 5.0% respectively[11]. The prevalence of GI bleeding (any) among patients infected with HIV who had ever been on ART was 8.9%; among patients with no record of ART use 6.4% had GI bleeding. As well, 0.1% of patients infected with HIV had a history of some type of ulcer. Using a comparator in the current study showed that these frequencies were significantly higher among patients infected with HIV relative to an HIV-uninfected population.

The 1.9% prevalence of PUD among patients infected with HIV in this study fits into the lower range of the published literature, which varies broadly from 0.44% to 15.4%[2,5,9,17,18]. One study assessed the rate of gastric and duodenal ulcers by the cART era and reported that the prevalence of gastric and duodenal ulcers prior to the cART era (1991–1994) was 3.8% for both conditions; during the early cART era (1999–2002) the rates were 9.7% and 2.9%, respectively, and were 8.3% and 6.6%, respectively, during the recent cART era (2005–2008)[9]. This was a much smaller sample of patients (*n* = 227) who were undergoing GI endoscopy, thus selecting for patients with upper GI issues.

Evidence suggests that GI bleeding is relatively infrequent among patients infected with HIV[12]. Pre-cART data indicate a prevalence of GI bleeding ranging from 4.2% to 16%[9,10,19], while post-CART data show generally lower trends ranging from 0.9% to 14.4%[9–11]. In Europe, an analysis of patients infected with HIV with GI bleeding in Spain reported a frequency of 11.4% before the cART era, and 14.4% after cART introduction[10]. However, a review of 706 patients infected with HIV who underwent GI endoscopy in Belgium observed lower rates. Data from the pre-cART era (1991–1994) showed that 4.2% of patients had bleeding upper GI lesions; this rate was 3.8% during the early cART era (1999–2002), and 0.9% during the recent cART era (2005–2008)[9]. In a smaller study of 450 patients with AIDS without GI symptoms at baseline, the cumulative incidence of acute upper GI bleeding was 3% at 6 months and 6% at 14 months[2]. These estimates for the recent cART era are higher than we found using a longer study period from 2005–2013.

We found that lower GI bleeding was more prevalent than upper GI bleeding in both study groups, whereas previous studies have shown upper GI bleeding to be up to 3 times higher than lower GI bleeding among patients infected with HIV[12]. Other studies have shown a relatively low incidence of upper (3%) and lower (2%) GI bleeding in patients infected with HIV[20]. Two studies reported rates for upper GI bleeding specifically, and these were both under 1%[9,11], while 1 study reported a higher rate of 5% for lower GI bleeding[11]. Some researchers have found that lower GI bleeding accounts for approximately 25% of all GI bleeding among patients infected with HIV[21]. In another survey, upper GI bleeding specifically accounted for 10% of all consultations over a 6-year period among patients infected with HIV attending a gastroenterology service at a US hospital[12]. However, information on ART use for this patient population was not available. In a survey of 156 patients infected with HIV who underwent endoscopy in an inner-city US hospital (pre-cART era), 16% were reported to have upper GI bleeding[19].

Effect measure modification using our hazards modeling criteria resulted in numerous significant EMMs in each model that would challenge interpretability with sparsely populated strata. Therefore, we made the ad hoc decision to combine comorbid conditions and comedications into composite EMMs, and then proceed with adjustment for confounding factors. After considering these EMMs and adjusting for confounders, the GI bleeding/PUD event rate was higher among patients infected with HIV with neither comorbidities nor comedications relative to HIV-negative subjects, but significantly decreased within the patient group that had both categories of risk factors (Table 3). This suggests that in the absence of other risk factors (no comedications, no comorbidities) HIV infection may play a larger role in increasing GI bleeding or PUD rates. However, in the presence of other comorbidities and comedications known to be associated with these outcomes, HIV status may not contribute as strongly to the development of GI bleeding and/or PUD.

Patients infected with HIV in the “with comorbidities and comedications” cohort stratum experienced a significantly lower relative rate of GI/PUD events. This unlikely result could be due to any combination of the following factors: (1) patients infected with HIV in general are more closely monitored and interact with clinicians regularly, resulting in higher diagnosis and prescription rates for comorbidities/coinfections (and possible treatment and/or prevention of GI symptoms) compared with HIV-negative subjects; (2) GI diagnoses are inconsistently diagnosed and managed among patients infected with HIV; (3) patients infected with HIV who are well controlled by cART and with few or milder coinfections address GI issues before they become too serious (unlike in [2]); or (4) patients infected with HIV take more over-the-counter medications and don’t seek physician care for GI issues compared with HIV-negative subjects. A limitation of collapsing variables into composite terms is that it is difficult to discern whether comedications or comorbidities are driving the results in this stratum.

Our study demonstrated that among patients experiencing a GI bleeding/PUD event, *H*. *pylori* was more prevalent among HIV-infected subjects compared with HIV-uninfected subjects. However, this diagnosis is not always captured due to a lack of or inconsistent testing. Studies suggest there is a lower prevalence of *H*. *pylori* in patients with HIV infection compared with *H*. *pylori* infection in HIV-negative subjects[22]. In the general population, prevalence of *H*. *pylori* in the adult population can be up to 65%, with 70% to 100% of patients with chronic gastritis and *H*. *pylori* infection also having PUD[23]. In contrast, in studies of HIV-positive individuals, the observed prevalence of *H*. *pylori* in cases of PUD has ranged widely from 0% to 49%[24–26].

There were a few variables our study was unable to capture fully. First, patients infected with HIV were not required to belong to a plan that submitted laboratory values, resulting in about a 1% sample with regards to CD4 counts and viral load; severity of HIV disease was an unknown factor that could have influenced the between-study differences in GI disorder prevalence estimates. In this small sample size, the majority of patients with lab data available were well controlled on ART, but missing data could be a result of systematic bias based on a lack of insurance coverage among the patients infected with HIV. This study was limited to individuals with commercial health or Medicaid coverage. Second, aspirin exposure, a potential risk factor for GI bleeding/PUD, was not fully classifiable due to over-the-counter use. A recent US nationwide survey of adults (aged 45–75 years) found that 52% reported daily aspirin use; 47% among 2039 respondents without a history of cardiovascular disease[27]. While higher doses of aspirin and aspirin-containing medications that would require a prescription are captured in this study, GI consequences of chronic low doses (not captured in this study) cannot be ruled out and remain as missing information.

Antiretroviral therapy exposure was not a significant predictor of GI/PUD in this study. Only about 56% of patients had any exposure to ART in this database, which is a likely underestimate attributable to other ways to access ART, such as the Ryan White Program (http://hab.hrsa.gov/abouthab/aboutprogram.html), which annually provides services and therapy to over 500,000 patients infected with HIV without adequate coverage in the United States. Although it is evident that ART can be associated with GI symptoms, it is difficult to isolate the contribution of a particular drug. Patients receive different drugs simultaneously while on cART, and our study demonstrated that this is often in addition to many non-ART prescriptions (and potentially unaccounted for over-the-counter medications).

Additional limitations to this study include that there was the possibility for coding inaccuracies in the diagnoses or procedures, as well as missing information on risk factors such as smoking, severity of HIV disease or other comorbidities, and missing information on over-the-counter medications associated with gastrointestinal disturbances.

Causes of GI symptoms are multifactorial and could stem from manifestations of HIV disease, such as opportunistic infections and visceral neuropathy characterized by gastroparesis and dietary habits[7]. Additionally, since GI symptoms are relatively common and are generally mild, many patients do not undergo routine evaluation when they occur; thus it is difficult to fully characterize the extent of the occurrence of these symptoms[28]. A database that is able to combine claims data with comprehensive HIV care information (survey or HIV-specific cohorts) would be better suited to fully address ART exposures and GI/PUD risk. More research is needed to examine the relationship between important comorbidities/coinfections, comedications such as aspirin and NSAIDs, and rates of GI bleeding and PUD in the HIV-infected patient population.

This study captured a large sample size of patients infected with HIV allowing for multivariate adjustment. Proceeding through the modeling strategy, we identified possible effect modification by recent comedication exposure and comorbidities—the components to these variables differing based on the outcome (GI bleeding, PUD, or both). Future research should examine the possibility that rates are differential based on classification of these strata; not controlling for EMM could result in biased estimates. We used a comprehensive adjustment set within our models, but, as with any observational study, uncontrolled confounding variables and/or missing data could have biased our findings.

In summary, rates of any GI bleeding, PUD, and both events comparing HIV-infected populations to non–HIV-infected populations were differential based on comorbidity and comedication status. Peptic ulcerative disorder rates, after stratifying on history of ulcers, were not significantly different based on HIV status. Antiretroviral therapy did not appear to be strongly associated with the risk of having a GI/PUD event, but was likely underestimated due to the under/uninsured population receiving ART from other sources.

## References

[1] Hill A, Balkin A. Risk factors for gastrointestinal adverse events in HIV treated and untreated patients. AIDS Rev. 2009;11(1):30–38. 19290032

[2] Parente F, Cernuschi M, Valsecchi L, Rizzardini G, Musicco M, Lazzarin A, et al Acute upper gastrointestinal bleeding in patients with AIDS: a relatively uncommon condition associated with reduced survival. Gut. 1991;32(9):987–990. 1916503 10.1136/gut.32.9.987 PMC1379034

[3] Bhaijee F, Subramony C, Tang SJ, Pepper DJ. Human immunodeficiency virus-associated gastrointestinal disease: common endoscopic biopsy diagnoses. Patholog Res Int. 2011;2011:247923 doi: 10.4061/2011/247923 21559197 10.4061/2011/247923 PMC3090068

[4] Bini EJ, Micale PL, Weinshel EH. Risk factors for rebleeding and mortality from acute upper gastrointestinal hemorrhage in human immunodeficiency virus infection. Am J Gastroenterol. 1999;94(2):358–363. doi: 10.1111/j.1572-0241.1999.858_a.x 10022629 10.1111/j.1572-0241.1999.858_a.x

[5] Mönkemüller KE, Call SA, Lazenby AJ, Wilcox CM. Declining prevalence of opportunistic gastrointestinal disease in the era of combination antiretroviral therapy. Am J Gastroenterol. 2000;95(2):457–462. doi: 10.1111/j.1572-0241.2000.01768.x 10685750 10.1111/j.1572-0241.2000.01768.x

[6] Chiu HM, Wu MS, Hung CC, Shun CT, Lin JT. Low prevalence of *Helicobacter pylori* but high prevalence of cytomegalovirus-associated peptic ulcer disease in AIDS patients: comparative study of symptomatic subjects evaluated by endoscopy and CD4 counts. J Gastroenterol Hepatol. 2004;19(4):423–428. 15012780 10.1111/j.1440-1746.2003.03278.x

[7] Chubineh S, McGowan J. Nausea and vomiting in HIV: a symptom review. Int J STD AIDS. 2008;19(11):723–728. doi: 10.1258/ijsa.2008.008244 18931263 10.1258/ijsa.2008.008244

[8] Karus D, Raveis VH, Alexander C, Hanna B, Selwyn P, Marconi K, et al Patient reports of symptoms and their treatment at three palliative care projects servicing individuals with HIV/AIDS. J Pain Symptom Manage. 2005;30(5):408–417. doi: 10.1016/j.jpainsymman.2005.04.011 16310615 10.1016/j.jpainsymman.2005.04.011

[9] Nkuize M, De Wit S, Muls V, Arvanitakis M, Buset M. Upper gastrointestinal endoscopic findings in the era of highly active antiretroviral therapy. HIV Med. 2010;11(6):412–417. doi: 10.1111/j.1468-1293.2009.00807.x 20146733 10.1111/j.1468-1293.2009.00807.x

[10] Olmos M, Araya V, Pskorz E, et al Coinfection: helicobacter pylori/human immunodeficiency virus. *Dig Dis Sci*. 2004;49(11–12):1836–1839. 15628713 10.1007/s10620-004-9580-5

[11] Vannappagari V, Nkhoma E, St Laurent S, Bowlin S. Prevalence of gastrointestinal bleeding in a cohort of HIV patients from a large US healthcare claims database. Abstract TUPE0090. Published at: 18th International AIDS Conference; July 18–23, 2010; Vienna, Austria.

[12] Chalasani N, Wilcox CM. Gastrointestinal hemorrhage in patients with AIDS. AIDS Patient Care STDS. 1999;13(6):343–346. doi: 10.1089/apc.1999.13.343 10842854 10.1089/apc.1999.13.343

[13] Balderas V, Spechler SJ. Upper gastrointestinal bleeding in a patient with AIDS. Nat Clin Pract Gastroenterol Hepatol. 2006;3(6):349–353; quiz following 353. doi: 10.1038/ncpgasthep0497 16741554 10.1038/ncpgasthep0497

[14] Kelly P, Saloojee H, Chen JY, Chung RT. Noncommunicable diseases in HIV infection in low- and middle-income countries: gastrointestinal, hepatic, and nutritional aspects. J Acquir Immune Defic Syndr. 2014;67(suppl 1):S79–S86. 25117963 10.1097/QAI.0000000000000260 PMC4159720

[15] Charpignon C, Lesgourgues B, Pariente A, Nahon S, Pelaquier A, Gatineau-Sailliant G, et al Peptic ulcer disease: one in five is related to neither *Helicobacter pylori* nor aspirin/NSAID intake. Aliment Pharmacol Ther. 2013;38(8):946–954. doi: 10.1111/apt.12465 23981105 10.1111/apt.12465

[16] Najm WI. Peptic ulcer disease. Prim Care. 2011;38(3):383–394, vii. doi: 10.1016/j.pop.2011.05.001 21872087 10.1016/j.pop.2011.05.001

[17] Werneck-Silva AL, Prado IB. Dyspepsia in HIV-infected patients under highly active antiretroviral therapy. J Gastroenterol Hepatol. 2007;22(11):1712–1716. doi: 10.1111/j.1440-1746.2007.04897.x 17559368 10.1111/j.1440-1746.2007.04897.x

[18] Chong VH, Lim CC. Human immunodeficiency virus and endoscopy: Experience of a general hospital in Singapore. J Gastroenterol Hepatol. 2005;20(5):722–726. doi: 10.1111/j.1440-1746.2005.03793.x 15853985 10.1111/j.1440-1746.2005.03793.x

[19] Bashir RM, Wilcox CM. Symptom-specific use of upper gastrointestinal endoscopy in human immunodeficiency virus-infected patients yields high dividends. J Clin Gastroenterol. 1996;23(4):292–298. 8957733 10.1097/00004836-199612000-00011

[20] Wallace MR, Brann OS. Gastrointestinal manifestations of HIV infection. Curr Gastroenterol Rep. 2000;2(4):283–293. 10981025 10.1007/s11894-000-0020-1

[21] Szajerka T, Jablecki J. Upper gastrointestinal bleeding in a young female with AIDS: a case report. Int J STD AIDS. 2012;23(3):e33–34. doi: 10.1258/ijsa.2009.009074 22581893 10.1258/ijsa.2009.009074

[22] Nevin DT, Morgan CJ, Graham DY, Genta RM. *Helicobacter pylori* gastritis in HIV-infected patients: a review. Helicobacter. 2014;19(5):323–329. doi: 10.1111/hel.12131 24773336 10.1111/hel.12131

[23] Nielsen H, Andersen LP. Serodiagnosis of Helicobacter pylori infection in patients with human immunodeficiency virus infection. APMIS. 1995;103(10):689–692. 8534426

[24] Blecker U, Keymolen K, Levy J, Vandenplas Y. Low prevalence of *Helicobacter pylori* in the acquired immunodeficiency syndrome. Am J Gastroenterol. 1993;88(8):1294 8338111

[25] Cacciarelli AG, Marano BJ Jr, Gualtieri NM, Zuretti AR, Torres RA, Starpoli AA, et al Lower *Helicobacter pylori* infection and peptic ulcer disease prevalence in patients with AIDS and suppressed CD4 counts. Am J Gastroenterol. 1996;91(9):1783–1784. 8792698

[26] Edwards PD, Carrick J, Turner J, Lee A, Mitchell H, Cooper DA. *Helicobacter pylori*-associated gastritis is rare in AIDS: antibiotic effect or a consequence of immunodeficiency? Am J Gastroenterol. 1991;86(12):1761–1764. 1660218

[27] Williams CD, Chan AT, Elman MR, Kristensen AH, Miser WF, Pignone MP, et al Aspirin use among adults in the U.S.: results of a national survey. Am J Prev Med. 2015;48(5):501–508. doi: 10.1016/j.amepre.2014.11.005 25891049 10.1016/j.amepre.2014.11.005

[28] Wilcox CM, Saag MS. Gastrointestinal complications of HIV infection: changing priorities in the HAART era. Gut. 2008;57(6):861–870. doi: 10.1136/gut.2006.103432 18203808 10.1136/gut.2006.103432

